# Transcriptional responses to proteotoxic stressors are profoundly diverse and tissue-specific

**DOI:** 10.1016/j.cstres.2026.100146

**Published:** 2026-01-29

**Authors:** Adelina Rabenius, Intisar Salim, Hilmar Lindström, Anastasiya Pak, Serhat Aktay, Anniina Vihervaara

**Affiliations:** Department of Gene Technology, KTH Royal Institute of Technology, Science for Life Laboratory, Solna, Stockholm, 171 65 Sweden

**Keywords:** Acute response, Chronic stress, Heat shock, Hsf1-/-, HSP90 inhibition, Huntington's disease

## Abstract

Cells counteract proteotoxic conditions by launching transcriptional stress responses. While synthesis of heat shock proteins (HSPs) upon acute stress is well characterized, how distinct proteotoxic conditions reshape the transcriptome remains poorly understood. Here, we analyse polyA+ RNA expression under heat shock, HSP90 inhibition, and polyglutamine (polyQ) aggregation. We find fundamentally distinct transcriptional responses to proteotoxic stressors and a systemic deficiency of mice under chronic stress to launch acute responses. While heat shock and HSP90 inhibition induce chaperones, polyQ aggregation increases expression of RNAs linked to transcription repression, chromatin remodeling, and autophagy. Analysing wild-type and Huntington's Disease (HD) mice reveals tissue-specific transcriptional adaptations to polyQ, including repressed cell-type specific functions and altered energy metabolism. Despite profound reprogramming, remarkably few genes exhibit consistently increased (*Acy3*, *Abhd1*, *Tmc3*) or decreased (*Fos*) RNA levels across HD brain regions. These results emphasize cellular background in disease manifestation and support energy metabolism and detoxifying enzymes as therapeutic targets in late-stage HD. Moreover, the systemic deficiency of chronically stressed mice to launch responses challenges strategies that rely on induced transcription. Altogether, we characterize transcription signatures to proteotoxic stresses, identify key *trans*-activators driving proteotoxic stress responses, provide an interactive gene-by-gene viewer of global changes, and delineate tissue-specific transcription programs in HD mice.

## Introduction

Proteotoxic stress challenges homeostasis and, if unmitigated, leads to accumulation of misfolded and aggregated proteins. To counteract stress, cells launch protective responses, such as heat shock response (HSR) in the cytosol and nucleus and unfolded protein response in the endoplasmic reticulum and mitochondria (reviewed in^1^). HSR is considered a rapid response to various stresses, including elevated temperatures, infections, heavy metals, protein aggregates, and chaperone incapacity (reviewed in^2^^,^^3^). However, the nature of distinct protein-damaging stresses can be profoundly different, requiring tailored responses. The most studied example, heat shock, triggers instant repression of transcription and translation, coupled to heat shock factor 1 (HSF1) driven activation of chaperone, co-chaperone, and polyubiquitin genes (reviewed in^4^). The produced chaperone machineries, in turn, hold misfolded proteins, refold them into correct conformation, prevent aggregation, and direct misfolded proteins to proteasomal degradation or autophagy (reviewed in^5^).

Neurons are particularly susceptible to unbalanced proteostasis,^2^ and thus, proteotoxic stress underlies neurodegenerative diseases such as Huntington’s Disease (HD). HD is a dominant genetic disease, inherited *via* abnormally long cytosine-adenine-guanine repeats in *Huntingtin* (*Htt*) gene^6^; (reviewed in^7^). The length of the encoded glutamine repeats (polyQ) varies in the mutant protein (mHTT), the longer stretches causing an earlier onset and more severe symptoms.^7^ The underlying mechanisms of how mHTT drives the disease are not fully understood. However, HD patients display protein aggregates in the brain, composed of mHTT and other proteins, including transcription factors, ubiquitin, proteasomal subunits, and chaperones. It remains unclear whether the aggregates drive the disease, are merely coincidental, or serve a protective role by sequestering more toxic intermediates (reviewed in^8^). Besides seeding protein aggregates, mHTT interacts with transcriptional regulators, causing epigenetic alterations and changing gene expression (reviewed in^9^).

Multiple studies have shown that the HSR is impaired in HD models and involves reduced activity or levels of HSF1,^10^, ^11^, ^12^, ^13^, ^14^ (reviewed in Gomez-Pastor et al.^15^ To restore HSR and HSF1 activity, small molecular drugs have been developed, including HSP90 inhibitors and HSF1 activators, giving various results in relieving HD symptoms (reviewed in Neef et al^16^). HSP90 is an essential ATP-dependent chaperone, one of the cell's most abundant proteins, and it interacts with a wide variety of clients.^17^, ^18^ The inhibitors disrupt HSP90′s ability to complete the chaperoning cycle, leading to increased tagging of clients to degradation.^19^ HSP90 inhibition also activates the HSR, which has been harnessed for therapeutic strategies.^21^, ^20^, ^16^ For instance, the HSP90 inhibitor HSP990^22^ was shown to induce HSF1 and temporarily relieve HD symptoms in mouse models. However, HSP990 did not give sustained results, likely due to the altered chromatin landscape impairing the HSR.^23^ In agreement, synthetic glucocorticoid dexamethasone downregulated HSP90, induced HSF1, and transiently relieved HD symptoms in mouse and fly.^13^ Also, direct activation of HSF1 *via* plant-derived Withaferin A ameliorated HD symptoms in mice.^24^ However, induced HSR *via* HSF1 activation or by inhibiting HSP90 has been reported to provoke an earlier appearance of mHTT-containing aggregates.^26^, ^25^ The various results and the consistent lack of long-lasting relieve manifest the difficulty to treat HD *via* small molecule inhibition. Moreover, the inability to ameliorate HD *via* induced HSR raises questions on how cells combat distinct proteotoxic stresses and which stress pathways could effectively mitigate polyQ stress.

To characterize cellular responses to distinct proteotoxic conditions, we compared transcription upon heat shock, HSP90 inhibition, and polyQ aggregation using existing information-rich RNA-seq datasets. First, we analyzed polyA+ RNA expression in muscle (*quadriceps femoris*) of three mouse genotypes: wild type (*WT*), HD model *R6/2*, and *Hsf1* knock-out (*Hsf1*^*-/-*^), all subjected to heat shock or HSP90 inhibition.^27^ The *R6/2* mouse expresses 115-150 Q repeats with a truncated (exon 1) mHTT, providing an excellent model for polyQ aggregation and fast HD progression.^28^ Next, we assessed tissue-specific reprogramming of RNA synthesis using data from the *Q175* HD mouse^29^ that contains 175 Q repeat in full-length mHTT and shows a late disease onset.^30^ Finally, we addressed age-derived changes by investigating RNA expression in the striatum of 2-month, 6-month, and 12-month-old *WT* and *Q175* mice.^31^ We found heat stress, HSP90 inhibition, and polyQ aggregation to launch profoundly different RNA expression programs. Moreover, mice under chronic stress of either HSF1 deficiency or polyQ expression displayed a systemic inability to mount acute responses. Through decades, HSF1-driven chaperone expression has provided a robust model for induced transcription^32^ and fostered key insights in proteostasis.^33^ However, focusing on this one conserved pathway has shadowed the complexity of transcriptional responses. We found clear *Hsp* induction only upon acute stresses of heat shock and HSP90 inhibition, whereas mice carrying polyQ aggregates responded *via* RNAs involved in autophagy, repression of transcription, and chromatin remodeling. Simultaneously, key metabolic pathways and tissue-specific processes were repressed. We conclude HSR to constitute a variety of stress-specific responses, driven *via* distinct *trans*-activators and tailored to the proteotoxic condition. We propose that full understanding of the profoundly different RNA expression programs, involving tissues-specific pathways and stress-specific protection, is crucial for designing therapeutic strategies and dissecting how cells combat adverse—acute or chronic—conditions. Finally, RNA expression programs across tissues highlight the importance of stage-dependent timing of HD treatments, and the lack of commonly induced or repressed genes suggest targeting pathways, such as energy metabolism, detoxifying enzymes, and autophagy, to ameliorate late-stage HD.

## Results

### Proteotoxic conditions trigger stress-specific RNA expression signatures

**Fig. 1:**
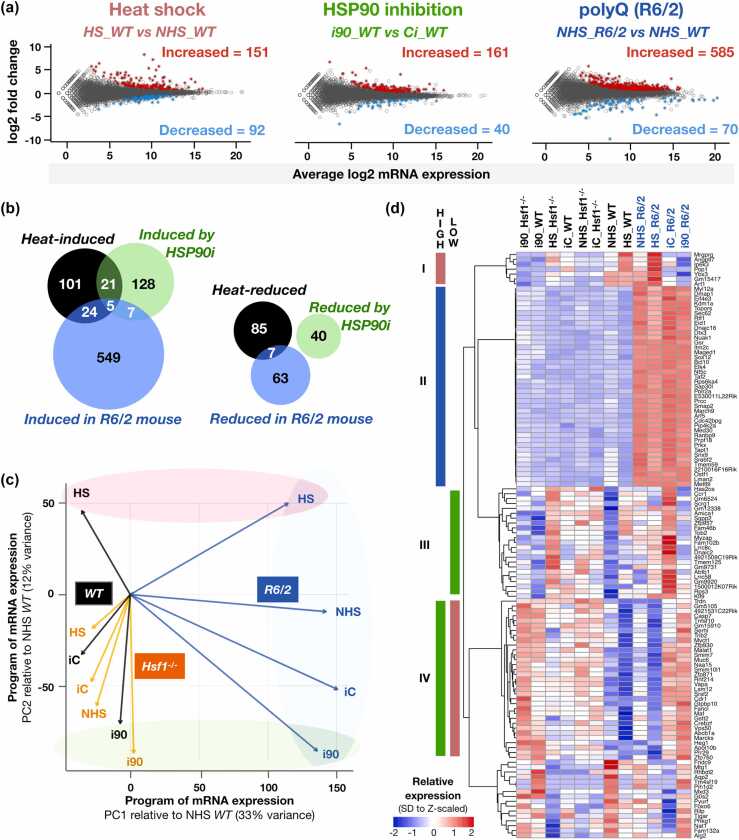
Heat shock, HSP90 inhibition, or polyQ aggregation launch distinct RNA expression programs. (a) MA-plots showing the average expression (x-axis) and stress-induced change (y-axis) of RNA expression. Left: heat shock (HS). Middle: HSP90-inhibition (i90). Right: polyQ expression (*R6/2*). Signiﬁcantly increased (red) and decreased (light blue) RNAs were identiﬁed with DESeq2 (*p*-value < 0.001 and |fold change| > 1.25. (b) Venn diagram depicting the number of signiﬁcantly induced (left) or repressed (right) genes. (c) RNA expression program in *WT* (black), *R6/2* (blue), and *Hsf1*^*-/-*^ (orange) mice under distinct proteotoxic stresses, projected to principal components 1 (x-axis) and 2 (y-axis). RNA expression program in non-stressed *WT* is positioned into the origo. The arrows show the direction and distance of transcription programs between phenotypes and conditions. PolyQ (*R6/2*), heat stress (HS), and HSP90 inhibition (i90) launch transcription programs into distinct directions, indicated with shaded blue, red, and green areas. (d) RNAs with top variance compared in a heatmap. Clusters I-IV contain RNAs with high (left) or low (right) expression in HS, HSP90i, or *R6/2*, colored as the shading in panel C**.**

To characterize transcriptional responses to distinct proteotoxic stresses, we analyzed RNA expression in muscle of *WT*, *R6/2*, and *Hsf1*^*-/-*^ mice exposed to heat shock or HSP90 inhibition.^27^ The heat shock *in vivo* was a whole-body heat pad for 20 minutes, followed by a 4-hour recovery (HS). For HSP90 inhibition, mice were treated with the small molecule HSP990 for 4 hours (i90). Respective control conditions were non-heat shocked (NHS) and vector control injected (iC) mice.^27^ We downloaded the polyA+ RNA-seq libraries (Figure S1A), mapped the raw data to the mouse genome (mm10), and ensured high replicate correlation and quality (Figure S1B). To find differentially expressed genes, we used DESeq2 that compares variance within replicates to the expression difference between conditions.^34^ Differential expression revealed that heat shock, HSP90 inhibition, and polyQ aggregation launched remarkably distinct RNA expression programs (Figure 1a and b). Only five genes showed statistically significant induction in all three proteotoxic stresses, while no gene was significantly repressed in all stress conditions (Figure 1b, Data S1). To understand the characteristics of transcription programs across genotypes and conditions, we performed principal component analysis (PCA), which projects high-dimensional data into lower dimensions, maximizing the retained variance. Transforming the expression programs into principal components (PCs) 1 and 2 confirmed that distinct proteotoxic stresses directed RNA expression in remarkably different directions (Figure 1c). While the polyQ-stressed *R6/2* mice were separated from *WT* and *Hsf1*^*-/-*^ mice along the PC1 (x-axis holding 33% of total variance), heat stress and HSP90 inhibition triggered cellular responses to opposite directions, visualized along the PC2 (y-axis, 12% of total variance). These results demonstrate the profoundly diverging responses depending on the proteotoxic condition.

To understand which genes contributed most to the variance in stress-induced programs, we selected the most important RNAs for PCs 1-3 and visualized their expression in a heatmap (Figure 1d). As expected from the PCA (Figure 1c), polyQ-expressing mice showed a distinguishable RNA expression program, with several genes induced specifically in *R6/2* (Figure 1d, group II). These genes were enriched with functions in negative regulation of RNA Polymerase II, histone phosphorylation coupled to DNA damage and chromatin remodeling, and lysosome activity (Data S2). A handful of genes (group I) were increased upon heat stress in *WT* and *R6/2*, but not in *Hsf1*^*-/-*^, mice (Figure 1D). The only chaperone or co-chaperone among the RNAs carrying most variance was *Dnajc16*, which was highly expressed in *R6/2* mice (Figure 1D) and regulates the size of autophagosome.^35^

### Acute stress induces chaperones - chronic polyQ stress activates autophagy

**Fig. 2:**
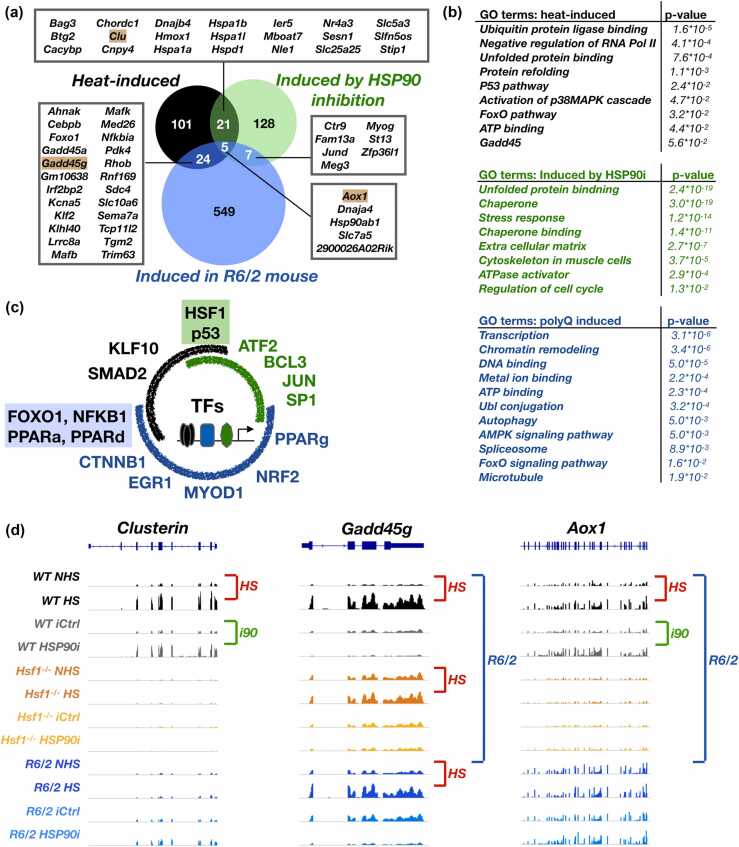
Heat stress and HSP90 inhibition induce chaperones, polyQ aggregation increases lysosome and autophagy-related RNAs. (a) Significantly increased RNAs compared in a Venn diagram. Genes that are induced in two or three proteotoxic stress conditions are listed in boxes. Genes indicated with golden background are shown in browser graphs in panel d. (b) Enriched GO terms among heat-induced, HSP90i-induced, and polyQ-induced genes. FDR-corrected p-value (Benjamini) is indicated for each category. (c) Enrichr analysis of transcription factors driving stress-specific transcription programs. Black, green, and blue arches indicate TFs associated with heat, HSP90i, and polyQ-induced RNAs, respectively. Overlapping arches and shaded text show factors associated with two stress conditions. Complete enrichr report is in Data S3. (d) Genome-browser examples of genes induced in a proteotoxic stress-specific manner. Clusterin (*Clu*) and *Gadd45g* mRNAs are induced in two conditions, *Aox1* is induced in all three conditions.

The profoundly different transcription programs in acutely (HS, HSP90i) and chronically (polyQ) stressed mice prompted us to seek genes that were induced (Figure 2a and b) or repressed (Figure S2b and c) across proteotoxic conditions. The five genes showing increased RNA expression in all three stresses encoded inducible HSP90 (*Hsp90ab1*) and co-chaperone DNAJA4, which has been described both as a heart-enriched co-chaperone for HSP70^36^; Hafizur et al,^37^) and a membrane-associated protein implied in cholesterol biosynthesis.^38^ The five RNAs commonly elevated across proteotoxic stresses also encoded solute carrier SLC7A5 that transports amino acids across membranes^39^ and Aldehyde oxidase 1 (AOX1), which neutralizes aldehydes and heterocyclic compounds.^40^

Beyond the five commonly induced genes, 21 were activated upon heat shock and HSP90 inhibition (Figure 2a). These genes encode canonical HSP70 (*Hspa1a*, *Hspa1b*, *Hspa1l1*) and HSP60 (*Hspd1*) foldases, extracellular chaperone (*Clu*), co-chaperones (*Bag3*, *Chordc1*, *Dnajb4*, *Stip1*), and other known stress-induced proteins (*Cacybp*, *Ier5*, *Hmox1*). Moreover, genes encoding solute carriers across plasma (*Slc5a3*) and mitochondrial (*Slc25a25*) membranes were induced upon heat shock and HSP90 inhibition. While chaperone induction was abundant upon the acute stresses of heat shock and HSP90 inhibition, only three genes for HSP chaperone complexes (*Hsp90ab1*, *St13*, and *Dnajc18*, an ER-localizing paralog of *Dnajc16*) were significantly activated in *R6/2* mouse (Data S1). The apparent lack of *Hsp* and *DnaJ* induction in muscle tissue of polyQ mice indicates that HD mice are either incapable of inducing chaperone machineries or use alternative pathways for maintaining proteostasis, likely due to polyQ aggregates being too large for chaperone-mediated clearance.^41^ Indeed, *R6/2* mice expressed significantly elevated levels of RNAs involved in autophagy (15 RNAs) and lysosome (21 RNAs) pathways (Figure 2b and Data S2), supporting polyQ aggregates to activate membrane-associated degradation, required for clearing large protein assemblies and damaged organelles.

### PolyQ-containing mice remodel energy metabolism and reduce muscle-specific RNAs

Inhibition of HSP90 is expected to disrupt proteostasis primarily *via* its cytosolic client proteins. In contrast, heat stress holds broad potential to instantly break intermolecular interactions, and polyQ aggregates cause a cascade of disrupted cellular functions. Intriguingly, HSP90 inhibition reduced expression of its largest client group, kinases,^17^ and increased expression of RNAs for protein-centric functions, including chaperones and cytoskeletal proteins (Figures 2b and S2B, Data S2). Heat stress and polyQ aggregation, instead, increased RNA expression for regulators of transcription, genome integrity, and metabolism, such as growth arrest and DNA damage inducible 45 (GADD45), Forkhead box O1 (FOXO1), and their known genomic targets (Figure 2a and b). Noteworthy is that the main repression in polyQ-expressing muscle involved muscle-specific pathways, including myofibril, muscle contraction, and sarcoplasmic reticulum (Figure S2b). The *R6/2* mouse also showed reduced levels of RNAs for essential metabolic processes, suggesting severely affected cell type-specific functions and cellular metabolism.

### Distinct sets of transcription factors drive proteotoxic stress responses

The different responses prompted us to analyze which transcription factors control the stress-specific changes in RNA expression. We performed Enrichr analyses^42^ on transcription regulatory relationships^43^ and, as expected, found HSF1 as the most prominent *trans*-activator for genes induced upon heat shock and HSP90 inhibition (Figure 2c, Data S3). Additionally, Tumor Protein 53 (p53), the coordinator of genome maintenance, cell cycle, and cancer suppression, was activated upon heat shock and HSP90 inhibition. No overlap on transcription factor activation was found upon HSP90 inhibition and polyQ expression. Instead, genes that exhibited increased RNA expression both in *R6/2* mice and upon heat shock were enriched for genomic targets of FOXO1, nuclear factor kappa B precursor, and peroxisome proliferator activated receptors (PPARs) (Figure 2c, Data S3). Nf-KB is a key regulator of inflammatory responses^44^, while FOXO1 is activated by nutrient deprivation and regulates metabolism and autophagy.^45^, ^46^ Furthermore, targets for all the main PPAR-family members (PPAR-a, PPAR-d, PPAR-g), which coordinate fatty-acid oxidation and energy metabolism (reviewed in^47^), were induced in *R6/2* mice (Figure 2c).

For stress-repressed genes, no shared transcription factor was identified (Figure S2c). In polyQ-expressing muscle, the downregulated genes contained targets of Krüppel like factor 3, which functions primarily as a transcription repressor and controls key muscle genes.^48^, ^49^ Additionally, genomic targets of Sterol regulatory element binding factor 1 (SREBF1) and Carbohydrate responsive element binding protein (ChREBP) were repressed (Figure S2c). While ChREBP promotes conversion of excess carbohydrates to triglycerides,^50^ SREBF1 controls cholesterol and fatty acid synthesis.^52^, ^51^ These changes in RNA expression lend strong evidence that *R6/2* mice reduce glycolysis (repressed ChREBP targets), experience nutrient deprivation (FOXO1 activation), suppress fatty-acid synthesis (repression of SREBF1), and activate fatty-acid oxidation and lipid catabolism (activated PPAR targets) as an alternative energy source. Altogether, the adapted RNA expression in *R6/2* mouse points to a profound shift in energy metabolism, from lipid biosynthesis to lipid catabolism.

### 3D stress cube: Gene-specific analyses of global responses

**Fig. 3:**
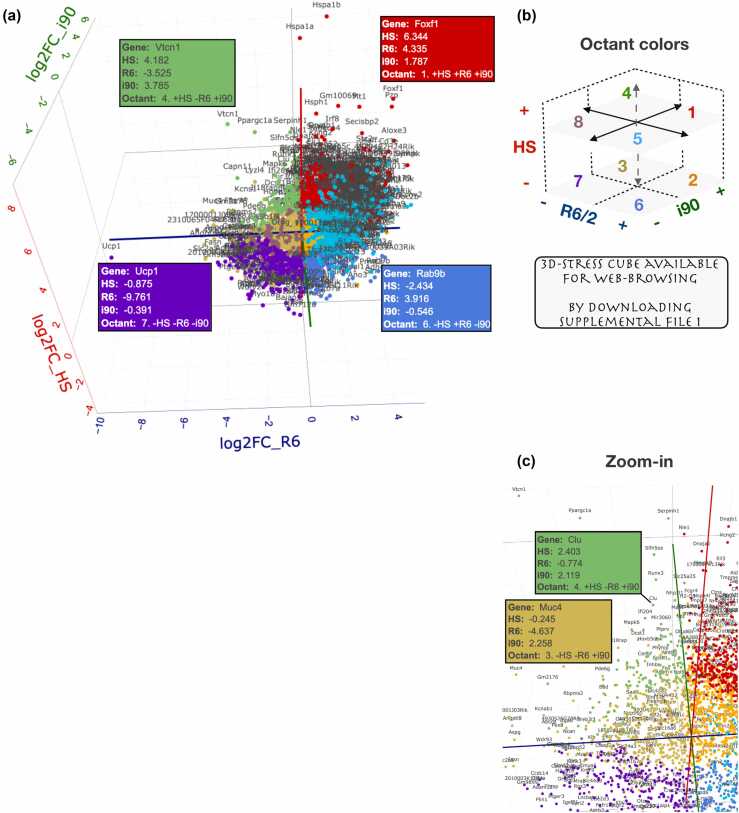
3D Stress Cube: Interactive visualization of stress-induced transcriptional changes. (a) Interactive 3D viewer shows transcriptional changes upon heat shock (HS), HSP90 inhibition (i90), and polyQ aggregation (R6). The RNAs are colored based on the octant, i.e., the direction of the stress-induced changes, schematically shown in panel b. *Vtcn1* (green), *Ucp1* (purple), *Foxf1* (red), and *Rab9b* (matt blue) are highlighted as RNAs in the distinct octants, and the direction (- or +) and log2FC in each stress condition shown in the infobox. (b) Color key for the octants. See Supplemental Figure 3 for example RNAs in octants 2, 3, 5, and 8. The 3D Stress cube is available for browsing by downloading and unzipping the File S1 (github.com/Vihervaara/3D-Stress-Cube) and opening the .html file in a web browser. (c) The cube allows zooming into specific areas, the boxes with RNA-specific information appear by placing the cursor over the dot. The 3D stress cube was generated with plotly,^53^ and stress-induced changes were counted as follows. Heat shock: log2(HS_WT/NHS_WT). HSP90i: log2(i90_WT/iC_WT). PolyQ: log2(NHS_R6/NHS_WT). The interactive cube contains polyA+ RNAs with combined log2FC >1.5 (*n* = 3496), counted as sqrt(log2FC_HS^2 + log2FC_R6^2 + log2FC_i90^2) >1.5. Data S4 contains all transcripts and their stress-induced expression changes.

DEseq2 uses strict statistical criteria to call differentially expressed genes, which could miss key genes and patterns in RNA expression programs. To provide gene-centric views within the context of the global changes, we generated an interactive 3D visualization tool, termed 3D Stress Cube (Figure 3 and File S1). This easy-to-use tool positions each RNA along x, y, and z axes according to log2 fold changes (log2FC) upon heat shock, HSP90 inhibition, and polyQ expression (Figure 3a). Each RNA is colored based on the directions of stress-induced changes (octants, Figure 3b), allowing instant identification of gene-by-gene changes within transcriptional responses. As examples, expression of *Foxf1* (octant 1, red) and *Ucp1* (octant 7, purple) RNAs were increased and decreased, respectively, in all three stress conditions (Figure 3a). RNAs with stress-specific responses are exemplified with *Vtcn1*, *Rab9b*, *Nppc*, *Angptl8*, *Odf3l2*, and *Ddit4* (Figure 3a and S3). These analyses showed 17% of RNAs to be increased and 8% to be decreased in all stresses (Data S4, all expressed RNAs, *n* = 11,757), which indicate slightly higher and lower occurrences than expected with a random change (12.5%). For RNAs with a total log2FC > 1.5 (Figure 3a, *n* = 3496), the fraction of RNAs induced in all stresses increased to 25%, while the fraction of RNAs repressed in all conditions remained around 8%. The cube is ready-to-use after downloading the File S1 in github.com/Vihervaara/3D-Stress-Cube and opening the html file in a web browser. Rotation, zooming, and information on each RNA (Figure 3c) are provided. For manageable browsing, the cube contains genes that show a minimum of 1.5 total log2FC across the three stresses (*n* = 3496). Data S4 lists all expressed genes and their responses.

### Acute responses are systemically hampered in mice under chronic stress

**Fig. 4:**
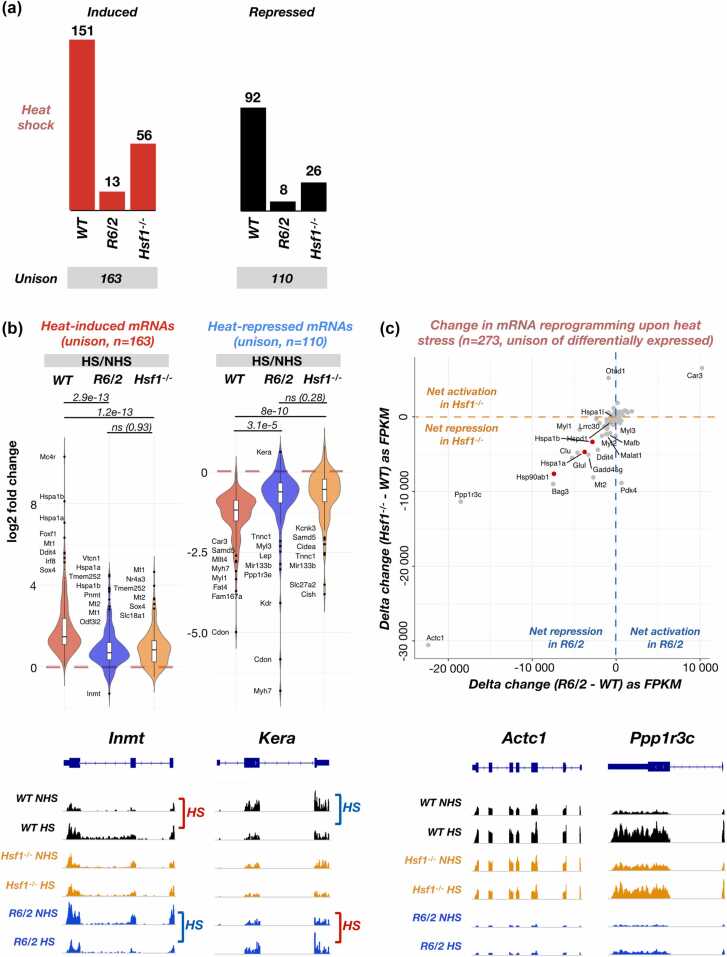
Heat shock response is systemically blunted in mice under chronic stress. (a) Number of differentially expressed RNAs upon heat shock in *WT*, *R6/2*, and *Hsf1*^*-/-*^ mice. Unison of induced (left) and repressed (right) RNAs are indicated below the bar charts. Related Supplemental Figure 4a contains MA-plots for *R6/2* and *Hsf1*^*-/-*^ mice. (b) Upper: Log2 fold change of differentially expressed RNAs upon heat shock, analyzed for the unison of induced (left) and repressed (right) RNAs in the three mouse genotypes. *P*-values from paired student’s *t*-tests are indicated for each pairwise comparison. Lower: Genome browser visualization of RNA expression from two outlier genes, *Inmt* and *Kara,* showing rare examples of changed directionality of stress response in *R6/2* mouse. (c) Upper: Acute stress response compared in *WT versus* chronically stressed (*R6/2* or *Hsf1*^*-/-*^) mice. Delta change in FPKM is derived as HS - NHS and compared as *R6/2* - *WT* (x-axis) or *Hsf1*^*-/-*^ - *WT* (y-axis). Lower: Genome browser examples of two outlier genes, *Actc1* and *Ppp1f3c*.

**Fig. 5:**
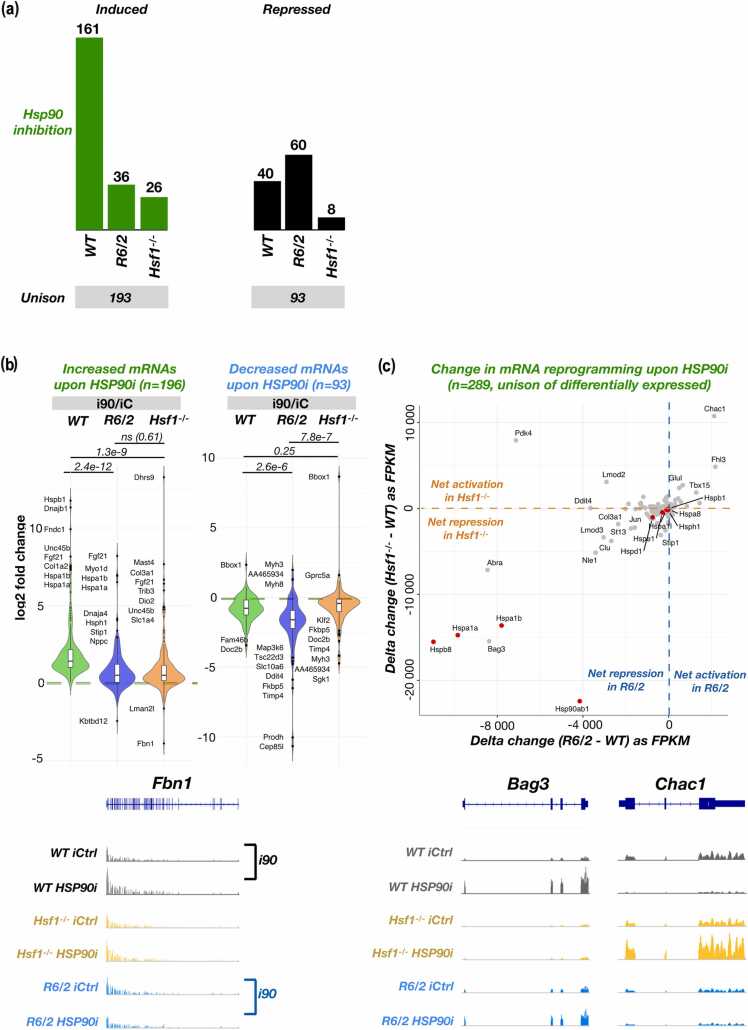
Transcriptional response to HSP90 inhibition is systemically blunted in mice under chronic stress. (a) Number of differentially expressed RNAs upon HSP90 inhibition in *WT*, *R6/2*, and *Hsf1*^*-/-*^ mice. Unison of induced (left) and repressed (right) RNAs are indicated below the bar charts. See related Supplemental Figure 4b for MA-plots of *R6/2* and *Hsf1*^*-/-*^ mice. (b) *Upper:* Log2 fold change of differentially expressed RNAs upon HSP90 inhibition, analyzed from the unison of induced (left) and repressed (right) RNAs in the mouse genotypes. P-values from paired student’s *t*-tests are indicated for each pairwise comparison. *Lower:* Genome browser example of an outlier gene, *Fbn1*. (c) *Upper:* Acute stress response in *WT versus* chronically stressed (*R6/2* or *Hsf1*^*-/-*^) mice. Delta change in FPKM is derived as HSP90i - Ci and compared as *R6/2* - *WT* (x-axis) or *Hsf1*^*-/-*^ - *WT* (y-axis). *Lower:* Genome browser examples of two outlier genes, *Bag3* (left) and *Chac1* (right).

To understand how mice under chronic stress mount transcriptional responses, we measured polyA+ RNA expression in *R6/2* and *Hsf1*^*-/-*^ mice subjected to heat shock (Figure 4) or HSP90 inhibition (Figure 5). While *R6/2* mice are under chronic stress due to polyQ aggregation, *Hsf1*^*-/-*^ mice have impaired metabolic pathways,^54^, ^55^ indicative of chronic stress. Moreover, *Hsf1*^*-/-*^ cells and mice are highly sensitive to induced stress.^56^, ^57^ DESeq2 analyses showed a drastic reduction in differentially expressed genes upon heat shock in *R6/2* and *Hsf1*^*-/-*^ mice (Figures 4a, 5a, and S4), as compared to *WT* mice (Figure 1a). These results are well in line with the original analysis by Neueder et al,^27^, where blunted responses in polyQ mice were reported. Importantly, the limited stress responses included global induction and repression and extended well beyond HSF1 target genes (Figures 1a and b, 4a and b).

Since fold changes can be large for genes with a low RNA expression, we compared gene-by-gene expression as difference (stress-control) between *WT* and chronically stressed mice (Figures 4c and 5c). This gene-centric comparison revealed a striking similarity between *R6/2* and *Hsf1*^*-/-*^ mice, as genes with reduced induction in polyQ mice were also less induced in mice that lacked HSF1 (Figures 4c and 5c). Given the different transcription programs in *Hsf1*^*-/-*^ *versus R6/2* mice (Figure 1c and d), these results indicate a systemic inability to mount acute transcriptional responses in chronically stressed mice, whether due to polyQ aggregates or lack of HSF1. This inability extended to induction and repression and occurred both upon heat shock (Figure 4) and HSP90 inhibition (Figure 5). Consequently, severely and broadly dampened ability to mount acute stress responses emerged as a feature of chronically stressed mice, not a limited inactivity of a single transcription activator or a pathway.

### Transcriptional changes to polyQ expression are tissue-specific

**Fig. 6:**
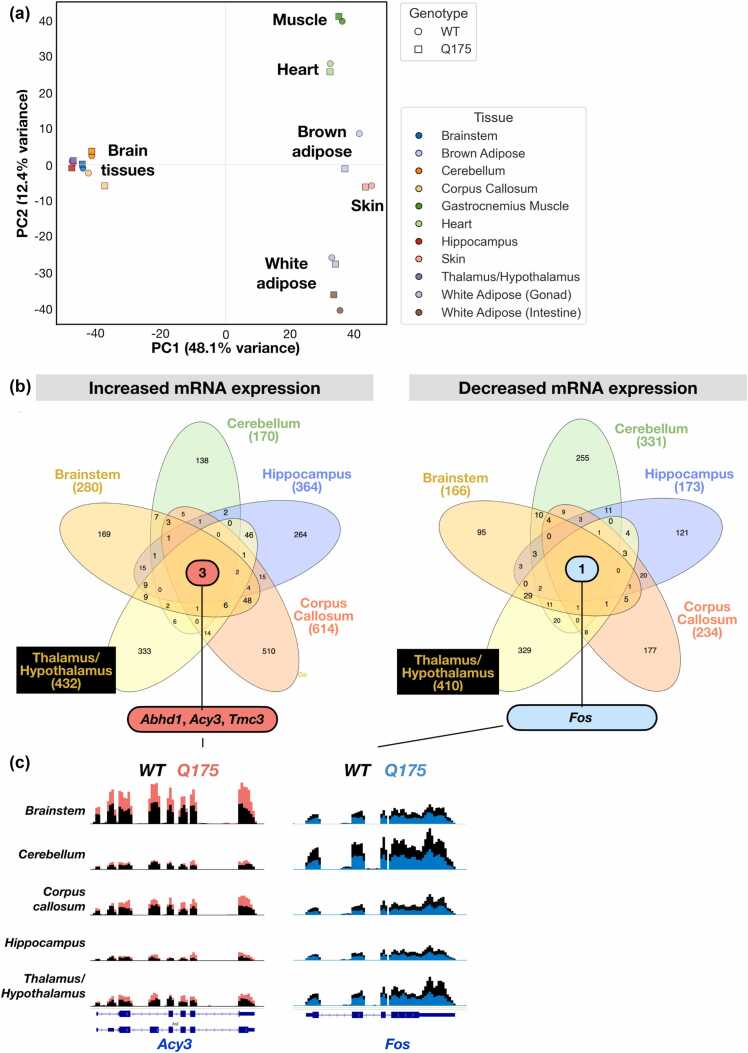
PolyQ stress causes tissue-specific reprogramming of RNA expression. (a) PCA analysis of RNA expression across tissues of *WT* and *Q175* mice. (b) Comparison of differentially increased (left) and reduced (right) RNAs between *WT* and *Q175* mice across tissues. Differential expression was analyzed with DESeq2 using *P*-value < 0.05 and |log2FC| > 0.5 as thresholds. The thee RNAs that are differentially induced (*Abhd1, Acy3, Tmc*) and the one (*Fos*) that is differentially reduced across brain tissues are shown. (c) *Acy3* (left) and *Fos* (right) RNA expression across brain tissues.

The distinct transcription programs (Fig. 1, Fig. 2) and stress responses (Figures 3-5) in striatal muscle of *WT* and *R6/2* mice prompted us to examine RNA expression across tissues. For this, we downloaded raw RNA-seq data generated by the HDinHD consortium^29^; hdinhd.org) in 11 tissues of *WT* and *Q175* mice. Particularly, we examined whether polyQ aggregates caused similar transcriptional changes across tissues and whether the absence of the canonical HSP response to polyQ aggregation was a general feature of HD pathology. PCA revealed profoundly tissue-specific RNA expression in *WT* and *Q175* mice (Figure 6a). While brain tissues were clustered close to one another (PCs 1 and 2), skeletal and heart muscle were separated from adipose tissues and skin along the PC2 (Figure 6a). Importantly, differences in RNA expression programs between *WT* and *Q175* mice were minute as compared to the tissue-specific expressions (Figure 6a). These results indicate that the adaptation to polyQ aggregates occurred within tightly tissue-constrained transcriptional environments. Next, we zoomed into the brain regions and found each region to exhibit a separable transcription profile (Figure S5a). The RNA expression variance attributable to polyQ aggregates, however, remained very small (Figure S5a). Thus, transcriptional responses to polyQ aggregates were remarkably tissue-specific, even within the central nervous system. Accordingly, DESeq2 analysis found hundreds of differentially expressed genes in the brain regions of the *Q175* mouse, as compared to the *WT* mouse, but little overlap in polyQ-induced changes between tissues (Figures 6b, S5B). GSEA analysis of affected pathways revealed repressed genes in *Q175* mice to be enriched with cell type-specific functions and energy metabolism (Data S5). These results are well in line with the reduced expression of RNAs for muscle-specific functions and metabolic pathways in *R6/2* mouse muscle (Figures 2 and S3). Conversely, immune-related RNAs were upregulated in several brain regions (most notably brainstem and corpus callosum) and in adipose tissues, while they were suppressed in the heart and gastrocnemius muscle (Data S5). We did not find evidence for the canonical HSP-inducing stress response in any of the investigated tissues of *Q175* mouse (Data S5).

Across the brain regions analyzed, only three mRNAs (*Abhd1*, *Acy3*, and *Tmc3*) were consistently upregulated, and one (*Fos*), consistently downregulated in *Q175* mouse (Figure 6b and c). *Fos proto-oncogene* is a rapidly responding gene that encodes c-Fos, a component of a potent *trans*-activator, Activator protein 1 (AP1). In neurons, reduced c-Fos levels are associated with impaired synaptic input, and altered c-Fos expression has been reported in some HD models.^58^, ^59^ Among the consistently increased RNAs, *Aminoacylase 3* (*Acy3*) encodes an enzyme involved in detoxification, capable to hydrolyze acylated amino acids generated as metabolites of protein degradation.^60^ α/β-hydrolase fold containing 1 (*Abhd1*) is likely an enzyme with lysophilic lipase activity, and it is involved in stress responses.^61^, ^62^ Transmembrane channel like 3 (*Tmc 3*) encodes a poorly characterized ion channel, expected to function in mechanosensing.^63^ Notably, *Abhd1* was previously reported to be upregulated in an HD cell model,^64^ which together with its increased expression in brain regions of *Q175* mouse could reflect tissue-wide compensatory mechanisms to maintain lipid homeostasis. Although *Abhd1* and *Tmc3* emerge as potential markers and influencers of HD pathology, both genes partially overlap with a strongly and seemingly ubiquitously expressed gene (*Preb* and *Gm16638*, respectively*)* in the mouse genome (Figure S5C). These overlaps call for strand-specific quantification of RNA expression to ensure correct quantification of RNA levels. To this end, *Acy3* and *Fos* provide robust and unambiguous examples of consistently increased and decreased RNAs across HD brain tissues (Figure 6c), suggesting their potentiality as HD markers and therapeutic targets. We conclude that similarities in responses to polyQ aggregates were more evident in induced and repressed pathways, rather than in differentially expressed genes. These findings underscore that transcriptional adaptation to polyQ toxicity is deeply tissue-specific and calls diagnostic and treatment strategies to target processes, such as energy metabolisms and lipid homeostasis, rather than individual proteins.

### Ageing changes RNA expression in mouse striatum but has a limited effect on *Hsp* levels

**Fig. 7:**
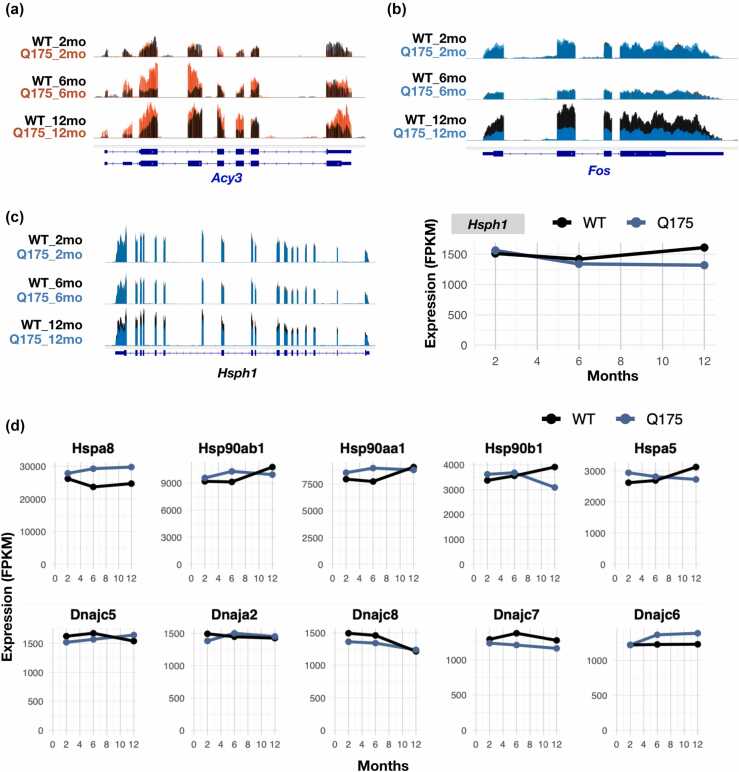
RNA expression changes in the striatum of aging *WT* and *Q175* mice. (a-c) Genome browser views on mRNAs whose expression is (a) increased (*Acy3)* and (b) decreased (*Fos*) in aging *Q175* mouse. (c) *Hsph1* mRNA expression shows a modest decline in the aging striatum of *Q175* mice. (d) Expression changes of the highest expressed *Hsp* (upper panels) and *Dnaj* (lower panel) mRNAs in the striatum of *WT* and *Q175* mice. For expression changes of all other *Hsp* mRNAs and most (FPKM > 200) *Dnaj* mRNA, see related Supplemental Figures S6B and S7A, respectively.

The cells' ability to respond to proteotoxicity changes with age. To analyze whether the lack of increased *Hsp* mRNA expression in polyQ mice was age-dependent, we re-mapped data by Diaz-Castro et al,^31^ generated in the striatum of *WT* and *Q175* mice of 2, 6, and 12 months of age. As reported in the original study,^31^ we identified several genes whose RNA expression was significantly increased or reduced in *Q175* mice during ageing. Intriguingly, these genes included the induction of *Acy3* (Figure 7a), *Tmc3* and *Abhd1* (Figure S6A), and repression of *Fos* (Figure 7b), further strengthening their potential as tissue-wide markers of HD. Comprehensive analysis of *Hsp* and *Dnaj* RNAs, instead, identified only modest differences in *WT versus Q175* striatum. These changes included reduced expression of *Hsph1* and increased expressions of *Hspa8* (HSC70) and *Dnajc18* mRNAs (Figure 7c and d, S7A). We found no clear pattern of induction or repression for components shared by chaperone complexes (Figures 7c and d, S6B and S7A) or *Hsf1* (Figure S7B) in ageing striatum. Based on RNA expression programs across tissues, we conclude that polyQ stress reduces expression of tissue-specific RNAs, alters energy metabolism, and has limited or varying effects on genes involved in canonical acute stress response. The analyses of RNA expression across tissues highlight energy metabolism pathways as potential targets and causes for impaired cellular functions in polyQ diseases. Moreover, the results suggest activities of key transcription factors, including FOXO1, GADD45, PPAR and Fos, and enzymes, such as ABHD1 and ACY3, as markers and contributors in HD pathology.

## Discussion

### The HSR comprises several stress-specific transcription programs

Since the discovery of heat shock proteins^65^ and HSF1 as their heat-induced *trans*-activator,^66^ analyses of stress responses have focused on this central and conserved pathway. As a result, many stress conditions that increase HSP expression, including heavy metals, infections, and neurodegenerative diseases, have been coined to induce the HSR.^2^ With the advent of techniques that identified all HSF1 binding sites or tracked transcription and RNA expression genome-wide, a remarkable complexity of HSF1 in controlling promoters and enhancers and driving distinct transcription programs in stress, cancer, and differentiation emerged.^67^, ^68^, ^69^, ^70^, ^71^, ^72^, ^73^, ^74^, ^75^, ^76^, ^77^, ^78^ In this study, we took a genome-wide approach to compare RNA expression changes triggered by distinct protein-damaging conditions. We found proteotoxic stresses, which all could be categorized under the broad term of HSR,^2^ to launch RNA expression programs to remarkably distinct directions (Figure 1). Only few genes were statistically significantly induced or repressed in two or three of the stress conditions (Figure 2). Even with loose criteria—analysing directions of the responses—less than a third of genes were either induced (25%) or repressed (8%) in all three proteotoxic conditions (Figure 3a and Data S4). We highlight that HSR should be considered as an umbrella term for a multitude of transcription programs that drastically differ from one another—each tailored to combat the specific adverse condition.

### Proteotoxic-specific responses occur *via* distinct set of transcription factors

Cellular stresses, such as oxidative stress, hypoxia, ER load, and temperature changes, activate dedicated stress-specific *trans*-activators (reviewed in^4^). However, no genome-wide comparison of transcription factor activities under distinct proteotoxic stresses had been performed. We found HSF1 and p53 targets to be induced upon acute stresses. Instead, the chronic stress of polyQ aggregation activated FOXO, PPAR, Catenin beta (CCNTB1), and Myoblast determination protein (MYOD1) targets and repressed lipid and glucose metabolism genes of SREBF1 and ChREBP (Figure 2). Altogether, the proteotoxic stress responses were driven *via* separate sets of transcription factors. The main overlap, i.e., the HSF1-activated chaperone network, covered less than 10% of induced genes upon heat shock or HSP90 inhibition (Figure 2). While acute stress induced chaperones, polyQ aggregates increased RNAs for autophagy and transcription repression, indicating a shift from RNA production to protein degradation. Moreover, polyQ expression repressed RNAs involved in tissue-specific functions and energy metabolism, further pointing to a broad slowdown in basal cellular processes in HD mice. Particularly, changing from glucose usage and storage to lipid catabolism (Figure 2 and S2) and disrupted mitochondrial processes (Data S5) indicate HD mice to struggle to meet the energy requirements. Worth noting is that chaperoning *via* foldases, such as HSP70, HSP90, TRiC, HSP10-HSP60, and HSP110, is highly ATP-consuming. Likewise, maintenance of the acidic environment in lysosomes and large-scale membrane remodeling in autophagy, requires considerable amount of energy. Taken together, the RNA expression changes across tissues of HD mouse manifest reduced basal operations, and attempt to maintain proteostasis *via* autophagy, likely in an environment with a limited energy supply.

### Impaired acute stress responses emerge as a hallmark of constitutive stress

Mice under chronic stress, either due to polyQ aggregates or lack of HSF1, displayed severely limited responses to acute stress (Fig. 4, Fig. 5). Intriguingly, RNA expression programs in HD mice suggest reduced RNA Polymerase II activity, a condition that ablates transcriptional responses to acute stress and differentiation.^79^ Many attempts have been made for ameliorating HD symptoms through restoring the HSR, which relies on the ability to launch a transcriptional response. Analyses of RNA expression programs in *R6/2* and *Q175* HD mice demonstrated profound challenges in inducing HSR in late-stage HD; First, the systemic deficiency to mount acute stress responses severely limits induced chaperone synthesis (Fig. 4, Fig. 5). Second, repression of RNA Polymerase II in HD mice (Figures 1 and 2 and S2, Data S5) could constitute the underlying cause for ablated responses and impair any *trans*-activator driven change. Third, no or low HSP increase was detected across polyQ-expressing tissue in *Q175* mice of distinct ages (Fig. 5, Fig. 6, Fig. 7, Data S5), pointing to cells preferring other means for restoring proteostasis in HD stages with solid-stage aggregates. These alternatively pathways could involve autophagy and detoxifying enzymes, such as ACY3 and AOX1 (Fig. 2, Fig. 6, Fig. 7). Fourth, the environment with altered metabolism and presumably reduced energy availability challenges activation of energy-heavy processes, including transcription, protein re-folding, and aggregate clearance. Indeed, support for energy production could aid HD cells to induce transcriptional responses and sustain cell-specific functions. We propose that systemic inability to mount acute stress responses is a hallmark of chronic stress and involves globally reduced transcription and disturbed energy balance.

### Emerging strategies for *mHtt* silencing, pathway targeting, and treatment timing

Recent years have witnessed an expansion of therapeutic strategies for HD treatment, including genome editing, neuroprotection, and transplantation of neuronal precursors. Targeting the root cause, i.e, removing or silencing *mHtt* holds the advantage of ameliorating early events of the disease. Accordingly, approaches that deliver RNAi, miRNAs, or CRISPR/Cas to target the *mHtt* are actively developed (reviewed in^80^). Of these, miRNA has proven successful in mice,^81^ and CRISPR/CasRx knockdown of *Htt* was recently reported.^82^ Also antisense oligos have been developed but faced challenges in clinical trials.^83^ While reducing *mHtt* acts on the early events, clinical strategies need combinatorial approaches with optimized timing to the HD stage. Indeed, mounting evidence shows induced polyQ expression to activate HSF1-driven HSPs expression and increased HSP levels to protect cells during early phases of polyQ pathology (reviewed in^84^). For example, hsf-1 activity and hsp expression ameliorate polyQ stress and extends lifespan in *Caenorhabditis elegans,*^26^, ^85^ which has a typical life span of 18-20 days. Growing evidence also shows certain chaperone complexes to maintain aggregation-prone intermediates in a soluble state,^87^, ^86^ and balanced co-chaperone networks to be critical in taupathies and HD.^88^, ^89^, ^90^ Once the polyQ inclusions mature into high-order solid state, however, HSPs likely fail to resolve the aggregation stress and could became trapped into the solid-state complexes. Mice have a typical lifespan of 2 years, and in the models investigated here, the solid-state aggregates had been formed. Accordingly, we did not find clear *Hsp* induction, but instead, uncovered broadly repressed cellular functions, altered metabolism, and increased autophagy. Hence, work by us and others emphasize the importance of timing and designing the treatment for the disease state; While reducing *mHtt* expression is efficient at early stages, HSP induction could handle soluble intermediates, ideally with tailored co-chaperone network. Once solid-stage aggregates form, management of collapsing cellular processes is required, and neuronal loss could be compensated with transplantation of neuronal precursors. To this end, the highly tissue-specific responses and disease progression during aging, challenge treating HD *via* individual proteins or pathways. However, the overarching defects in maintaining cellular processes across tissues raises possibilities for improving energy availability, RNA Polymerase function, lipid synthesis, and autophagy in late-stage HD with solid-state polyQ aggregates. Taken together, our results support stage-timed approaches that reduce *mHtt* expression, induce HSP networks, balance energy needs, improve autophagy, and boost enzymatic de-toxification in treating HD.

## Conclusions

We analyzed RNA expression programs under distinct proteotoxic conditions and found heat shock, HSP90 inhibition, and polyQ aggregation to launch fundamentally distinct transcriptional responses *via* different sets of *trans*-activators. Only acute stresses of heat stress and HSP90 inhibition induced chaperone production. Additionally, heat-stressed cells activated FOXO, GADD, and MAPK pathways, while HSP90 inhibition induced expression of RNAs for cytoskeletal and extracellular matrix proteins. In stark contrast, chronic stress of polyQ aggregation increased expression of RNAs involved in transcription repression, chromatin remodeling and autophagy. PolyQ aggregation also reduced the expression of tissue-specific RNAs and altered energy metabolism. Mice under chronic stress, whether due to HSF1 deficiency or polyQ aggregation, displayed a systemic inability to mount acute stress responses. In agreement, across brain and muscle tissues, and in mice of different ages, polyQ aggregation had limited effects to the expression of the canonical acute stress-induced RNAs. Our results highlight the importance of timing medical treatments to ameliorate polyQ aggregation diseases. To improve cellular functions in stages of solid-state polyQ aggregation, we propose targeting energy metabolism and autophagy pathways, rather than inducing individual proteins or acute stress responses.

## Materials and methods

### Obtaining and aligning RNA-seq data in *WT*, *R6/2*, *Q175*, and *Hsf1*^*-/-*^ mice

Raw RNA-seq data in *WT*, *R6/2*, *Q175*, and *Hsf1*^*-/-*^ mice was obtained from three studies and downloaded as fastq files from Gene Expression Omnibus. The obtained raw data was processed to sequencing depth normalized coverage files and gene expression counts. The polyA+ RNA-seq data from mouse *quadriceps femoris* under distinct stress conditions was obtained from^27^ (GSE95602). The polyA+ RNA-seq data in 11 tissues of *WT* and *Q175* mice originated from the HDinHD consortium (www.HDinHD.org^29^; GSE65775), and RNA-seq data in the striatum of 2-, 6-, and 12-month-old *WT* and *Q175* mice was from^31^ (GSE124846). From GSE124845 data, we downloaded the striatal RNA-seq (input). For each sample and replicate, fastq files were obtained with sra-tools (fasterq-dump), and the quality analyzed with fastqc (https://www.bioinformatics.babraham.ac.uk/projects/fastqc/). Where needed, the reads were trimmed to remove low-quality ends and filtered for high quality with fastx-tools (https://github.com/agordon/fastx_toolkit). The reads were mapped to mouse genome annotations (mm10 or GRCm39) with a splice-aware aligners HiSat2^91^ and STAR.^92^ The obtained.bam files were sorted with samtools^93^ and converted to bedgraph with bedtools^94^ and to bigWig with BedgraphToBigWig (https://www.encodeproject.org/software/bedgraphtobigwig/).

### RNA expression analysis

In each sample, RNA expression was measured with featureCounts^95^ or HTseq,^96^ counting alignments to exons for each transcript. The obtained RNA expression was compared between replicates by visualizing log2-converted RNA expressions in xy-graphs and analyzing statistical significances with Spearman rank correlation (rho). After ensuring high correlation, replicates were merged into coverage (bigWig) files, and the RNA expression of replicate-merged data visualized with Integrative genomics viewer (IGV).^97^ Within each IGV browser image, the y-scales of all tracks are identical and linear. Differential gene expression was assessed with DESeq2,^34^ which compares variance of expression within replicates to the expression difference between conditions. To call differential expression in the comparison of three protein-damaging stresses, adjusted *P* value < 0.001 and a minimum fold change 1.25 (1.25 for increased, 0.8 for decreased) were required. To account for the higher variation of RNA expression between tissues, adjusted *P* value < 0.05 and [log2FC] > 0.5 were used as thresholds. RNAs with less than 10 counts (average of replicates) were considered unexpressed. Differential RNA expression was visualized in MA-plots, showing RNAs with significantly increased expression in red and significantly decreased expression in light blue. Across the analyses of proteotoxic stresses, heat shock and HSP90 inhibition were matched to their respective controls, HS against NHS and i90 against iC. In each MA-plot, violin graph, and xy-plot, the exact comparison is indicated. Final RNA expression values are mean expression within replicates against kilobases of transcript length and sequencing depth (FPKM).

### Principal component analysis

PCA was performed with prcomp R package on log2 transformed RNA expression. In stress response analyses, RNA expression in all genotypes and conditions, including non-heat-shock (NHS), heat shock (HS), vector control (iC), and Hsp90-inhibition (i90) were included. The expression programs were transformed into PCs 1 and 2 that held 33% and 12%, respectively, of the total variance within all the RNA expression programs. In the xy-graph, each RNA expression program is represented as a dot, and the expression program of unstressed WT mouse (NHS_WT) placed into the origo. The arrows are displacement vectors (mean expression vectors) in the PCA space and show the direction and distance of each RNA expression program related to the RNA expression program in the NHS_WT mouse. In the analysis of RNA expression across tissues, log2 expression means in all *WT* and *Q175* tissues were included. Next, PCA was conducted with brain tissues only.

### Correlation matrices and heatmap

Top 40 most contributing RNAs for variance in PCs 1, 2, and 3 were selected (120 RNAs in total). The expression of the selected RNAs were z-scaled, and the scaled expression visualized on a heatmap generated with the pheatmap package in R. The names of the 120 RNAs are displayed on the heatmap, and the color scale indicates mean (white), high (red, positive z-score), and low (blue, negative z-score) RNA expression.

### Gene ontology analyses

Gene ontology enrichments within RNA groups were searched with DAVID tool^98^ using annotation clustering. The FDR-corrected p-values (Benjamini) are shown on the graphs and tables. Due to the very small group of GO terms within downregulated genes upon HSP90 inhibition, also kinases are included where the uncorrected *P* value, but not Benjamini, was significant (indicated with an asterisk). Differential gene expression in tissues of *WT* and *Q175* mice was analyzed with Gene Set Enrichment Analysis (GSEA).^99^ Full GO term reports are available in Data S2 (DAVID GO) and Data S5 (GSEA). To identify transcription factors that control genes in the selected groups, we used gene set knowledge discovery with enrichr,^42^ with transcription regulatory relationship unraveling TRUSST database^43^ with standard settings. Full enrichr reports are available in Data S3.

### Generation of interactive 3D graphs

Stress-induced changes in RNA expression were reported in log2FC as follows. HS: log2(HS_WT / NHS_WT); i90: log2(i90_WT/iC_WT), and R6: log2(NHS_R6/NHS_WT). The log2FC of each RNA was graphed in 3D, one axis per stress using R package plotly,^53^ which provides interactive web-based graphics for dynamic rotation, zooming, and point-inspection. The 3D vizualizations were saved as.html compatible files that can be loaded for interactive, user-guided examination by downloading and unzipping File S1 from github.com/Vihervaara/3D-Stress-Cube, and opening the.html file in a web browser. The points are colored based on octant (see schematic cube in Figure 3). The total distance of each RNA from the origo was counted as sqrt(log2FC_HS^2^ + log2FC_R6^2^ + log2FC_i90^2^). In the interactive visualization, RNAs with a min total distance 1.5 are shown. Data S4 lists all the RNAs and their transcriptional changes during proteotoxic stresses.

### Comparison of transcriptional responses in muscle of chronically stressed mice

After calling significantly induced or repressed genes in each mouse genotype, a unison of induced or repressed genes in a given stress condition was identified. RNA expression changes upon heat shock and HSP90 inhibition was, thereafter, analyzed in the unison of induced or repressed genes in the given condition. Violin graphs compare reprogramming of RNA expression in *WT*, *R6/2*, and *Hsf1*^*-/-*^ mice as log2FC. Statistical significance between genotypes is counted with paired student's *t*-test, showing the *P* value for each comparison on the violin graphs. Since fold change can be high for lowly expressed genes, we also compared differences in RNA induction and expression as total difference, FPKM_treatment - FPKM_control. We first counted the difference in each genotype (e.g., heat shock in WT = HS_WT - NHS_WT) and then compared the total difference between mutant and *WT* as Delta change (e.g., change in *R6/2* - change in *WT*). An example calculation for delta change in heat shock between *R6/2* and *WT* is: (HS_R6 - NHS_R6) - (HS_WT - NHS_WT). The delta change is compared across unisons of induced and repressed RNAs, and examples of outlier RNAs are shown in the genome browser.

## CRediT authorship contribution statement

**Adelina Rabenius:** Writing – original draft, Formal analysis, Conceptualization. **Intisar Salim:** Writing – review & editing, Visualization, Formal analysis, Data curation, Conceptualization. **Hilmar Lindström:** Writing – review & editing, Formal analysis. **Anastasiya Pak:** Writing – review & editing, Formal analysis. **Serhat Aktay:** Writing – review & editing, Formal analysis. **Anniina Vihervaara:** Writing – original draft, Supervision, Resources, Project administration, Methodology, Funding acquisition, Formal analysis, Data curation, Conceptualization.

## Declaration of generative AI and AI-assisted technologies in the writing process

No AI tool has been used in the writing of this manuscript.

## Declaration of interest

The authors declare that they have no known competing financial interests or personal relationships that could have appeared to influence the work reported in this paper.

## Data Availability

All data is publicly available and we make every effort to make our data, results, and code available for the scientific community.
